# Acute intraperitoneal infection with a hypervirulent *Acinetobacter baumannii* isolate in mice

**DOI:** 10.1038/s41598-019-43000-4

**Published:** 2019-04-25

**Authors:** Greg Harris, Rhonda KuoLee, H. Howard Xu, Wangxue Chen

**Affiliations:** 1 Human Health Therapeutics Research Center, National Research Council Canada, 100 Sussex Drive, Ottawa, Ontario K1A 0R6 Canada; 2 Department of Biological Sciences, California State University, Los Angeles, Los Angeles, CA 90032 USA; 3 Department of Biology, Brock University, St. Catharines, Ontario L2S 3A1 Canada

**Keywords:** Bacterial host response, Mouse

## Abstract

*Acinetobacter baumannii* infection has become a major cause of healthcare-associated infection and a critical pathogen in the WHO antimicrobial resistance research and development priority list. Catheter-related septicemia is one of the major clinical manifestations of *A. baumannii* infection associated with high morbidity and mortality. In this study, we used a clinical *A. baumannii* strain (LAC-4) that is hypervirulent to immunocompetent C57BL/6 and BALB/c mice and established a mouse model of intraperitoneal (i.p.) *A. baumannii* infection. Our study showed that i.p. LAC-4 infection of C57BL/6 and BALB/c mice induces a lethal or sublethal infection with high bacterial burdens in peritoneal cavity, blood and tissues and the infected mice either succumbed to the infection within 24 hours or completely recovered from the infection. The infection induces acute peritoneal recruitment of neutrophils and other innate immune cells, and the local and systemic production of proinflammatory cytokines and chemokines (IL-1β, IL-5, IL-6, TNF-α, RANTES, MIP-1β, MCP-1, KC and IL-10). Mechanistic studies suggest an important role of macrophages in the host innate defense in this model in that *in vitro* stimulation of peritoneal macrophages with killed LAC-4 induced a similar pattern of cytokine/chemokine responses to those in the infected mice, and depletion of peritoneal macrophages rendered the mice significantly more susceptible to the infection. Thus, this mouse infection model will provide an alternative and useful tool for future pathogenesis studies of *A. baumannii-*associated septicemia and identification and characterization of important virulence factors, as well as serve as a surrogate model for rapid evaluation of novel therapeutics and vaccines for this emerging infectious agent.

## Introduction

*Acinetobacter baumannii* is a major cause of hospital-acquired infections worldwide^1–4^. In addition, *A. baumannii* infections are increasingly difficult to treat due to its rapid development of resistance to multiple antibiotics^5,6^. Indeed, *A. baumannii* has recently been listed as a critical priority pathogen (Priority 1) for R&D of new antibiotics by the WHO^7^.

Catheter-related bacteremia and sepsis is one of the major manifestations and most severe forms of *A. baumannii* infection in hospital setting and the overall mortality of can be as high as 70%^1,8^. The mouse model of intraperitoneal (i.p.) *A. baumannii* infection has been commonly used for studying the infection pathogenesis and for evaluating novel therapeutics and vaccine efficacy^9–15^. However, to establish an effective infection and bacteremia in mice, most *A. baumannii* isolates will be required either to be admixed with porcine mucin prior to inoculation, or immunocompromised hosts such as neutropenic or diabetic mice must be used^4,12,16^. Despite the common use of mouse models of i.p. *A. baumannii* infection by the *Acinetobacter* research community, only a few studies have systemically characterized this model, particularly the host response to the infection^15,17^. Those studies showed that the infection induces production of interleukin (IL)-17 and IL-23 and neutrophils play an important role in host resistance to the infection, which is independent of the IL-17/IL-23 pathway^15^.

We have previously identified a clinical isolate of *A. baumannii* (LAC-4) that is much more virulent in mice when it was inoculated intranasally (i.n.)^18^ or intravenously (i.v.)^19^ as compared to most other clinical isolates and ATCC type strains of *A. baumannii*^20,21^, with significantly higher bacterial burdens in the lungs and enhanced extrapulmonary dissemination to the spleen^18^. Genomic analysis of the LAC-4 isolate reveals that it harbors unusually high copies of five insertion sequences and possesses a rare capsule biosynthesis locus contained within a genomic island of approximately 33 kb in length^22^. In this study, we evaluated the potential of this clinical isolate for its utility in a mouse model of intraperitoneal infection and characterized the innate host cellular and proinflammatory cytokine responses to the infection.

## Results

### Intraperitoneal infection of LAC-4 in C57BL/6 and BALB/c mice

**Figure 1:**
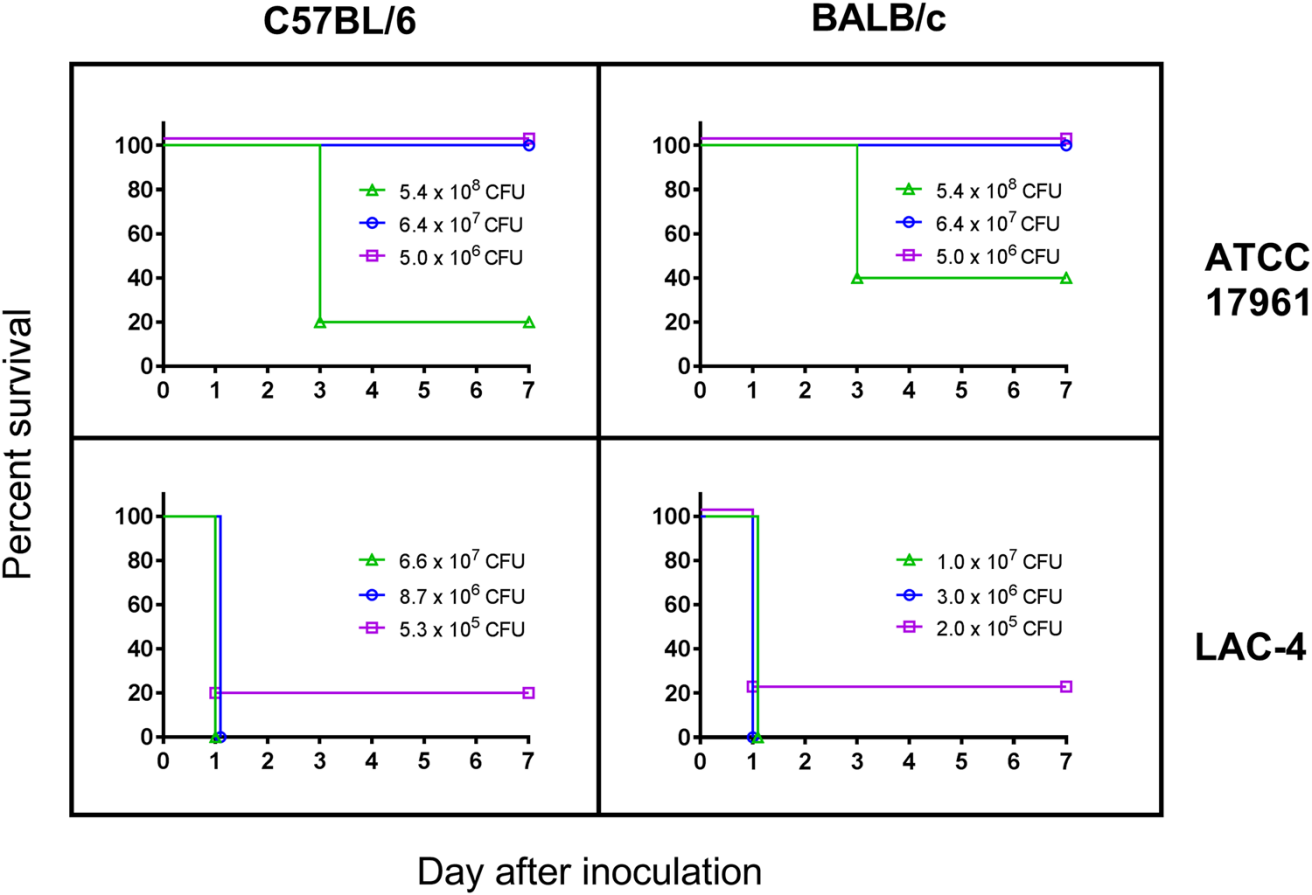
Surviving rates of C57BL/6 and BALB/c mice following i.p. inoculation with *A. baumannii* ATCC17961 (top panel) and LAC-4 (bottom panel). Groups of 5 C57BL/6 or BALB/c mice were i.p. inoculated with various numbers of ATCC17961 or LAC-4 as indicated and their clinical outcome monitored daily for 7 days. The number of surviving mice/total number of mice infected were 11/15 and 12/15 for C57BL/6 and BALB/c mice infected with ATCC17961, respectively, and 1/15 for C57BL/6 and BALB/c mice infected with LAC-4.

To determine if this strain is also hypervirulent in mice when inoculated intraperitoneally, we first compared survival rates of C57BL/6 and BALB/c mice after i.p. inoculation with varying doses of ATCC 17961 and LAC-4. All C57BL/6 and BALB/c mice succumbed to infection within 24 h after i.p. inoculation with 10^7^ or 10^6^ colony forming units (CFU) of LAC-4, and 80% of the either mouse strain succumbed to 10^5^ CFU of LAC-4 infection (Fig. 1, lower panel). In contrast, no mice died after i.p. inoculation with up to 6.4 × 10^7^ CFU ATCC 17961, but 80% of C57BL/6 and 60% of BALB/c mice succumbed to 5 × 10^8^ CFU of ATCC 17961 inoculation (Fig. 1, upper panel). Thus, LAC-4 is at least ~100 times more virulent than ATCC 17961 in both C57BL/6 and BALB/c mice when inoculated intraperitoneally, which is similar to the virulence difference between the two isolates when inoculated intranasally or intravenously^18,19^.

### Kinetics of *in vivo* bacterial growth after i.p. LAC-4 inoculation

**Figure 2:**
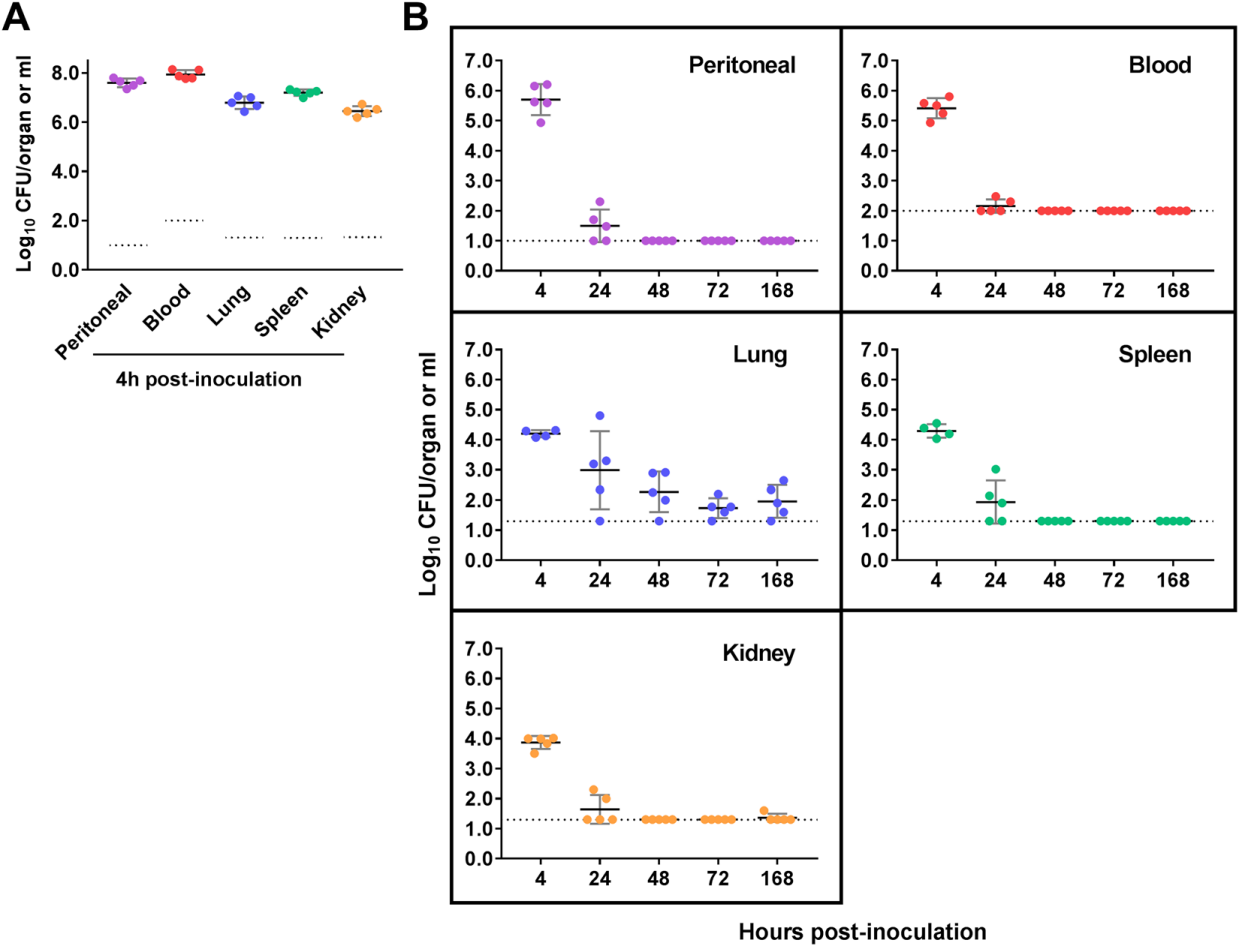
Bacterial burdens in the peritoneal cavity, blood, lungs, spleen, and kidneys of C57BL/6 mice following i.p. LAC-4 inoculation. Groups of C57BL/6 mice (n = 5) were i.p. inoculated with 1 × 10^7^ (**A**) or 5 × 10^4^ (**B**) CFU of *A. baumannii* LAC-4. Bacterial burdens in the peritoneal lavage fluid, blood and respective organs at various times post-inoculation were determined by quantitative bacteriology. Data from individual mouse are presented as scatter plot and the horizontal bar represents the mean. The results represent one of at least two experiments with similar results. The detection limits for bacterial burdens were indicated as black dotted lines (**A**,**B**).

To further characterize the host response to i.p. LAC-4 infection, groups of C57BL/6 mice were inoculated i.p. with either a lethal (10^7^ CFU) or a sublethal dose (5 × 10^4^ CFU) of LAC-4 and the kinetics of bacterial growth in the peritoneal cavity, lungs, spleen, kidneys and blood were determined. As shown in Fig. 2, regardless of the initial inoculum dose, LAC-4 cells were detected in blood and all tissues examined at 4 hours post inoculation (h p.i.). In mice inoculated with the lethal dose of LAC-4, the bacterial burdens in the peritoneal cavity and blood at 4 h p.i. were about 50-fold higher than the inoculum, and the bacterial burdens in the lung, spleen and kidney reached 10^7^ CFU per organ (Fig. 2A), indicating rapid local replication and systemic dissemination of LAC-4 after i.p. inoculation. All the mice succumbed to the infection before 24 h p.i. In mice inoculated with the sublethal dose of LAC-4, the bacterial burdens in the peritoneal cavity, blood, spleen and kidneys had decreased significantly at 24 h p.i. and were generally below the detection limit by 48 h p.i., with the exception in the lung where small numbers of bacteria were consistently cultured throughout the study period (168 h p.i.) (Fig. 2B).

### Inflammatory cell responses in the peritoneal cavity after i.p. LAC-4 infection

**Figure 3:**
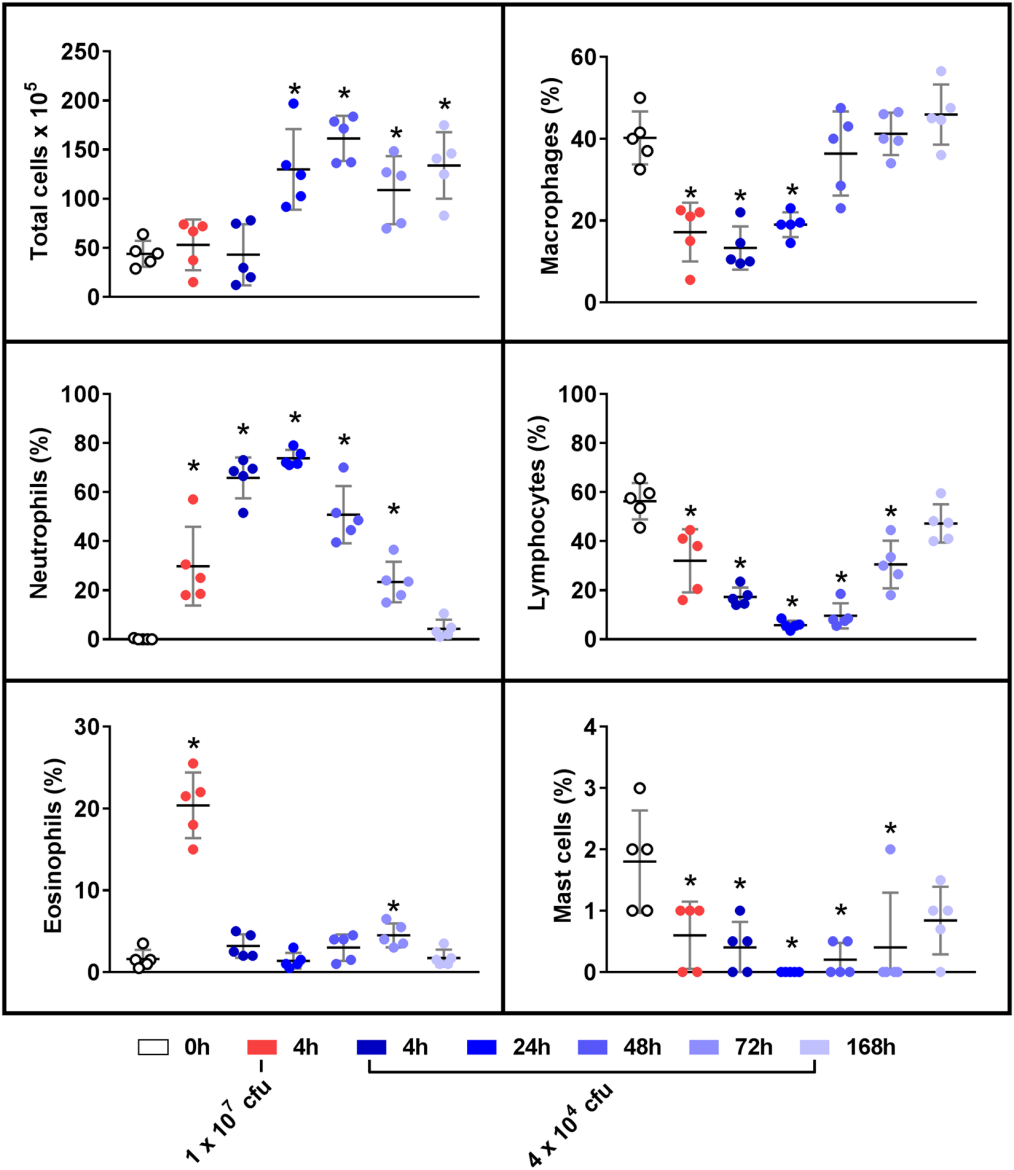
Peritoneal inflammatory cell responses in mice with a lethal or sublethal intraperitoneal infection of *A. baumannii* LAC-4. Groups of C57BL/6 mice (n = 5) were inoculated i.p. with a lethal (1 × 10^7^ CFU, 4 h only) or sublethal (5 × 10^4^ CFU) dose of LAC-4 and killed at 0, 4, 24, 48, 72 and 168 hours post-inoculation. The peritoneal cavity was lavaged, and the total cell counts were determined with a hemacytometer and differential cell counts were determined on cytospin slides (Cytospin 3, Shandon, Pittsburgh, PA) stained with Hema-3 (Fisher Scientific, Kalamazoo, MI). Data from individual mouse are presented as scatter plot and the horizontal bar represents the mean. The results are representative of at least two independent experiments with similar results. *p < 0.05 vs. 0 h group (One-way ANOVA with Dunnet’s multiple comparisons test).

To characterize the host innate immune responses to i.p. LAC-4 infection, total number of peritoneal lavage cells and their subpopulations from mice i.p. inoculated with a lethal (10^7^ CFU) or sublethal (5 × 10^4^ CFU) dose of LAC-4 were examined. As anticipated, macrophages and lymphocytes were the predominant types of peritoneal cells at 0 h, followed by mast cells and eosinophils. Neutrophils were almost absent at this time point (Figs 3 and S1). At 4 h p.i., the total number of peritoneal cells was not significantly changed in lethally infected mice, but the number and percentage of peritoneal neutrophils and, to a lesser extent, eosinophils were significantly increased (P < 0.05). Accordingly, the total number and particularly the percentage of peritoneal macrophages and lymphocytes were significantly decreased (Figs 3 and S1).

Similar to the lethally infected mice, the sublethally infected mice showed no significant change in the total number of peritoneal cells at 4 h p.i. (Figs 3 and S1), despite the presence of nearly 10^7^ CFU LAC-4 in the peritoneal lavage fluid at this time point (Fig. 2B). The total number of peritoneal cells in the sublethally infected mice was significantly increased at 24 h p.i., peaked at 48 h p.i. and remained significantly above baseline at day 7 (Figs 3 and S1). In addition, both the proportion and total number of peritoneal neutrophils were increased at 4 h p.i., peaked between 24 and 48 h p.i., and remained higher than baseline level by day 7 (Figs 3 and S1). Although the percentage of peritoneal macrophages was significantly reduced at 4 and 24 h p.i. as a result of increased neutrophil recruitment, the total number of macrophages was not significantly affected at those time points and was significantly increased thereafter (P < 0.05). Both the total number and percentage of peritoneal lymphocytes were significantly decreased at 4, 24 and 48 h p.i., began to recover at 72 h p.i. and returned to the baseline level at 168 h p.i. Although some statistically significant changes in the total numbers of eosinophils and mast cells were observed at some time points after the infection, their total numbers and associated changes were overall minor (Figs 3 and S1).

**Figure 4:**
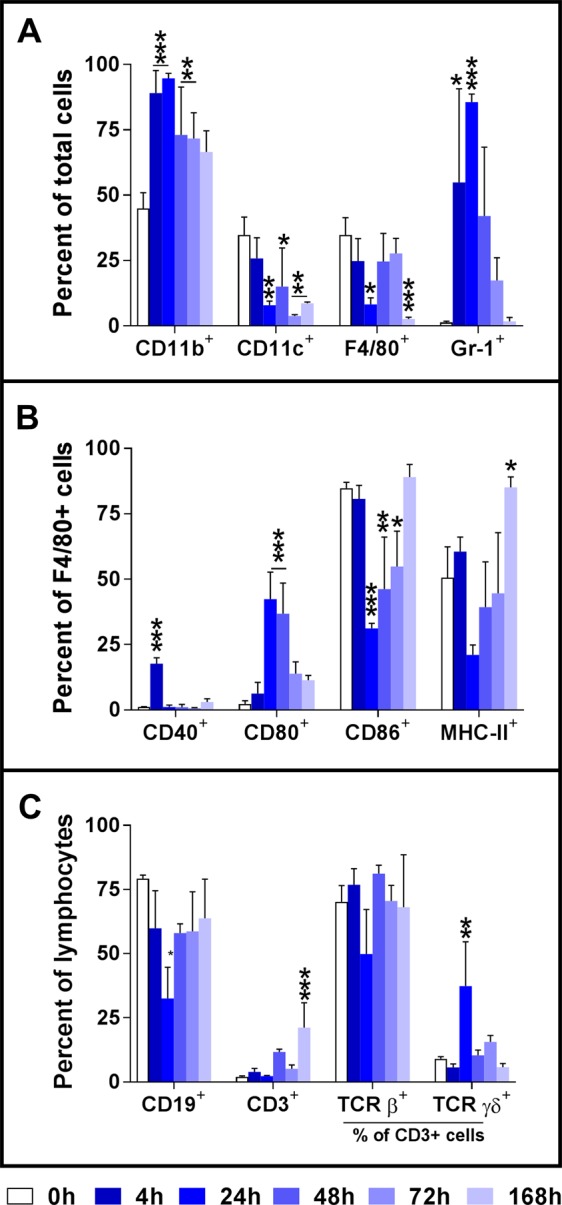
Activation of F4/80+ peritoneal macrophages following intraperitoneal inoculation with *A. baumannii* LAC-4. Groups of C57BL/6 mice were i.p. inoculated with 3.3 × 10^4^ CFU of freshly cultured LAC-4. Mice were euthanized at the indicated times and the peritoneal cavity was lavaged. The expression of CD11b^+^, CD11c^+^, F4/80^+^, Gr-1^+^, CD19^+^ and CD3^+^ cells (**A**,**C**) as well as the expression of CD40, CD80, CD86 and MHC-II by F4/80^+^ macrophages (**B**) and TCRβ and TCRγδ by CD3^+^ lymphocytes (**C**), measured as mean fluorescence intensity (MFI), was determined by FACS analysis. Data are compiled from two independent experiments (n = 3 for each experiment) with similar results, and are presented as mean ± SD of % positive cells. *p < 0.05; **p < 0.01; ***p < 0.001 vs 0 h (One-way ANOVA with Dunnet’s multiple comparisons test).

We also analyzed the phenotype changes of peritoneal cells in the sublethally infected mice over the course of the infection (0 to 168 h p.i.). A representative result from three independent experiments is shown in Fig. 4. The percentage of CD11b^+^ peritoneal cells was significantly increased between 4 and 96 h p.i. whereas the percentage of CD11c^+^ cells significantly decreased from 24 h p.i. and thereafter, and did not return to the baseline level at 168 h p.i., the end of our study (Fig. 4A). Consistent with the cytology data, the percentages of F4/80^+^ and Gr-1^+^ cells were significantly decreased or increased respectively; these changes peaked at 24 h p.i. (Fig. 4A). In addition, there was significant reduction in the percentage of F4/80^+^ cells at 168 h p.i. (Fig. 4A).

Analysis of the activation markers on F4/80^+^ peritoneal macrophages showed a significant increase in the percentage of CD80^+^ macrophages and decrease in the percentages of CD86^+^ or MHC-II expressing macrophages while the percentage of CD40^+^ macrophages generally showed no substantial changes over the course of the infection (Fig. 4B). As anticipated, the majority of the peritoneal lymphocytes are CD19^+^ B cells with small percentage of CD3^+^ T cells (Fig. 4C). The percentage of CD19^+^ lymphocytes decreased significantly at 24 h p.i. but returned almost to the baseline level by 48 h. On the other hand, there were no significant changes in the percentages of CD3^+^ cells until day 7 when its percentage increased significantly (Fig. 4C). Within CD3^+^ cells, overall there were no significant changes in the proportions of αβTCR^+^ or γδTCR^+^ cells, except that the percentage of γδTCR^+^ cells were significantly, but with large variations, increased at 24 h p.i. (Fig. 4C).

### Intraperitoneal LAC-4 infection induces potent local and systemic proinflammatory cytokine and chemokine responses

**Figure 5:**
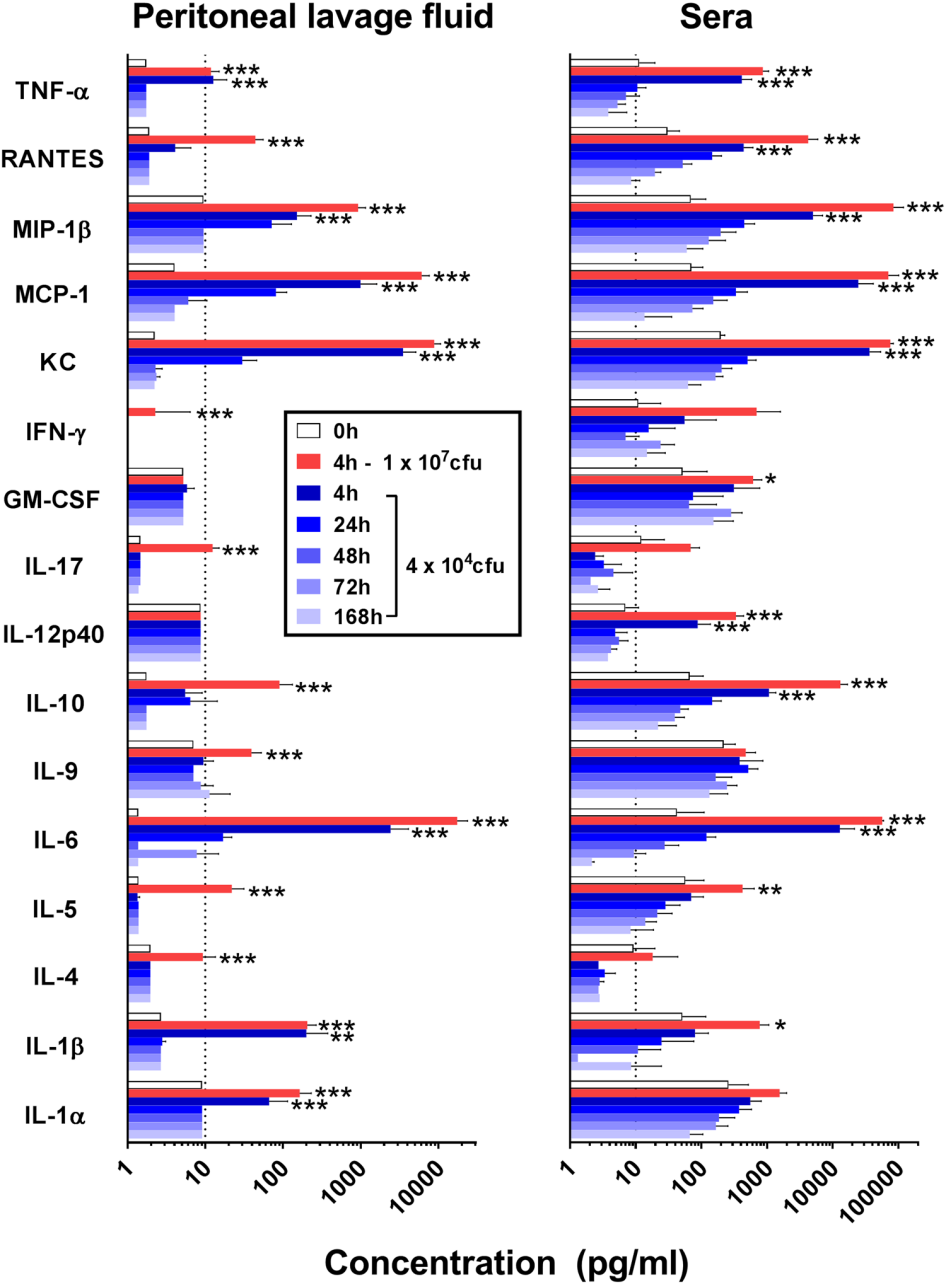
Cytokine and chemokine levels in the peritoneal lavage fluid and sera of C57BL/6 mice following lethal or sublethal i.p. LAC-4 infection. Groups of C57BL/6 mice (n = 5) were i.p. inoculated with 1 × 10^7^ (lethal) or 5 × 10^4^ (sublethal) CFU of LAC-4, and sacrificed at the indicated hours post-inoculation. Cytokine and chemokine levels were determined in the sera and peritoneal lavage fluid using 21-plex Milliplex mouse cytokine/chemokine kits on a Luminex 100IS system. The detection limit for all cytokines and chemokines is <10 pg/ml (dot lines). All values are mean ± SD. *p < 0.05; **p < 0.01; ***p < 0.001 vs 0 h (One-way ANOVA with Dunnet’s multiple comparisons test). The levels of IL-2, IL-3, IL12p70, IL-13, and VEGF were below <10 pg/ml in all samples assayed and they were not plotted in the graph for clarity purpose.

To further characterize the inflammatory and innate immune responses to i.p. LAC-4 infection and their potential implication in the infection pathogenesis, local (peritoneal lavage fluid) and systemic (serum) levels of a panel of 21 proinflammatory cytokines and chemokines were determined at various time points after infection; several of these have previously been implicated in the immunopathogenesis of *A. baumannii* infection in mouse models of i.p., i.v., and i.n. infections and in human cell culture studies^4,15,18,21,23^. Compared to 0 h, lethally infected mice showed significant increases in the levels of 14 out of the 21 chemokines/cytokines measured in the peritoneal lavage fluid at 4 h p.i. (Fig. 5) whereas the sublethal infection induced a transient elevation in the level of IL-1α, IL-1β, IL-6, KC, MCP-1, MIP-1β and TNF-α in the peritoneal lavage fluid; these levels peaked at 4 h p.i. and generally returned to the baseline level by 48 h p.i. (Fig. 5).

Similarly, i.p. inoculation of mice with a lethal dose (10^7^ CFU) of LAC-4 induced significant elevation of serum levels of GM-CSF, IL-10, IL-12p40, IL-1β, IL-5, IL-6, KC, MCP-1, MIP-1β, RANTES, and TNF-α at 4 h p.i. (Fig. 5). Mice receiving a sublethal dose (5 × 10^4^ CFU) of LAC-4 i.p. inoculation showed significant increases in the serum levels of IL-6, IL-10, IL-12p40, KC, MCP-1, MIP-1β, RANTES and TNF-α only at 4 h and almost all those elevated cytokines/chemokines returned to the baseline levels by 48 h (Fig. 5). There were no significant changes in the serum levels of IFN-γ, IL-4 or IL-9, or any of the other analytes tested (IL-2, IL-3, IL-12p70, IL-13, and VEGF) at any time point, regardless of the inoculation doses used. These results suggest that the sublethal i.p. infection with LAC-4 is a relatively acute and severe one but the host is able to control the infection and clear the infection rapidly and efficiently under normal circumstance.

### *In vitro* cytokine/chemokine responses by peritoneal macrophages to killed LAC-4

**Figure 6:**
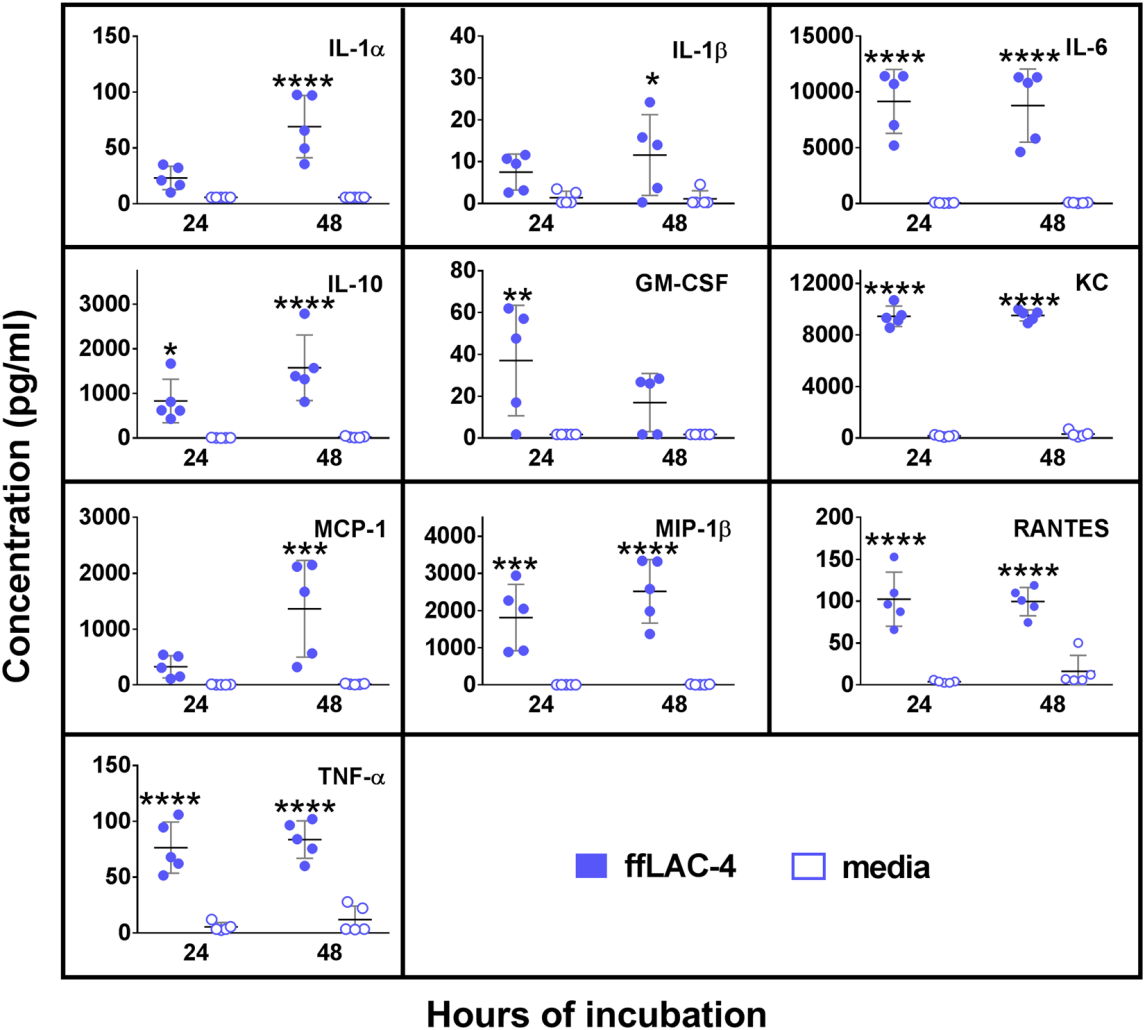
Proinflammatory cytokine and chemokine responses by formalin-fixed LAC-4 (ffLAC-4)-stimulated peritoneal macrophages. Peritoneal lavage cells were harvested from 8–10 wk-old, female C57BL/6 mice (n = 5) and 5 × 10^5^ cells were incubated with ffLAC-4 (MOI = 100) or culture media alone. Medium (DMEM) was supplemented with 10% FBS, 5.5 µM 2-Mercaptoethanol, 25 mg/ml 4-(2-hydroxyethyl)-1-piperazineethanesulfonic acid (HEPES) and non-essential amino acids (MEM-NEAA). Cell culture supernatants were collected at 24 and 48 h. The concentrations of indicated cytokines/chemokines were measured using a 21-plex Milliplex mouse cytokine/chemokine kit on a Luminex 100IS system. The detection limit for all cytokines and chemokines is <10 pg/ml. Data are presented as scatter plot of duplicate culture wells, and the horizontal bar represents the mean. *P < 0.05, **P < 0.01, ***P < 0.001 vs media group (Two-way ANOVA with Sidak’s multiple comparisons test).

Macrophages are one of the predominant residential innate immune cells in the peritoneal cavity. Previous studies by us and others have shown that macrophages play important roles in the early host defense against *A. baumannii* and other Gram-negative bacterial pathogens^15,19,24,25^. To further understand the interaction between LAC-4 and peritoneal macrophages, we examined the chemokine and cytokine responses to *in vitro* formalin-fixed LAC-4 (ffLAC-4) stimulation by cultured peritoneal macrophages (Fig. 6). In corroboration with the *in vivo* serum and peritoneal lavage fluid data from the infected mice (Fig. 5), the levels of IL-6, IL-10, KC, MIP-1β, RANTES, and TNF-α were significantly increased in the culture supernatant of peritoneal macrophages at both 24 and 48 h whereas the GM-CSF level increased at 24 h only and the levels of IL-1α, IL-1β, and MCP-1 increased at 48 h only. Levels of IL-2, IL-3, IL-4, IL-5, IL-9, IL-12p40, IL-12p70, IL-13, IL-17, IFN-γ and VEGF remained unchanged at both time points (data not shown).

### Role of peritoneal macrophages in resistance to i.p. LAC-4 infection in mice

**Figure 7:**
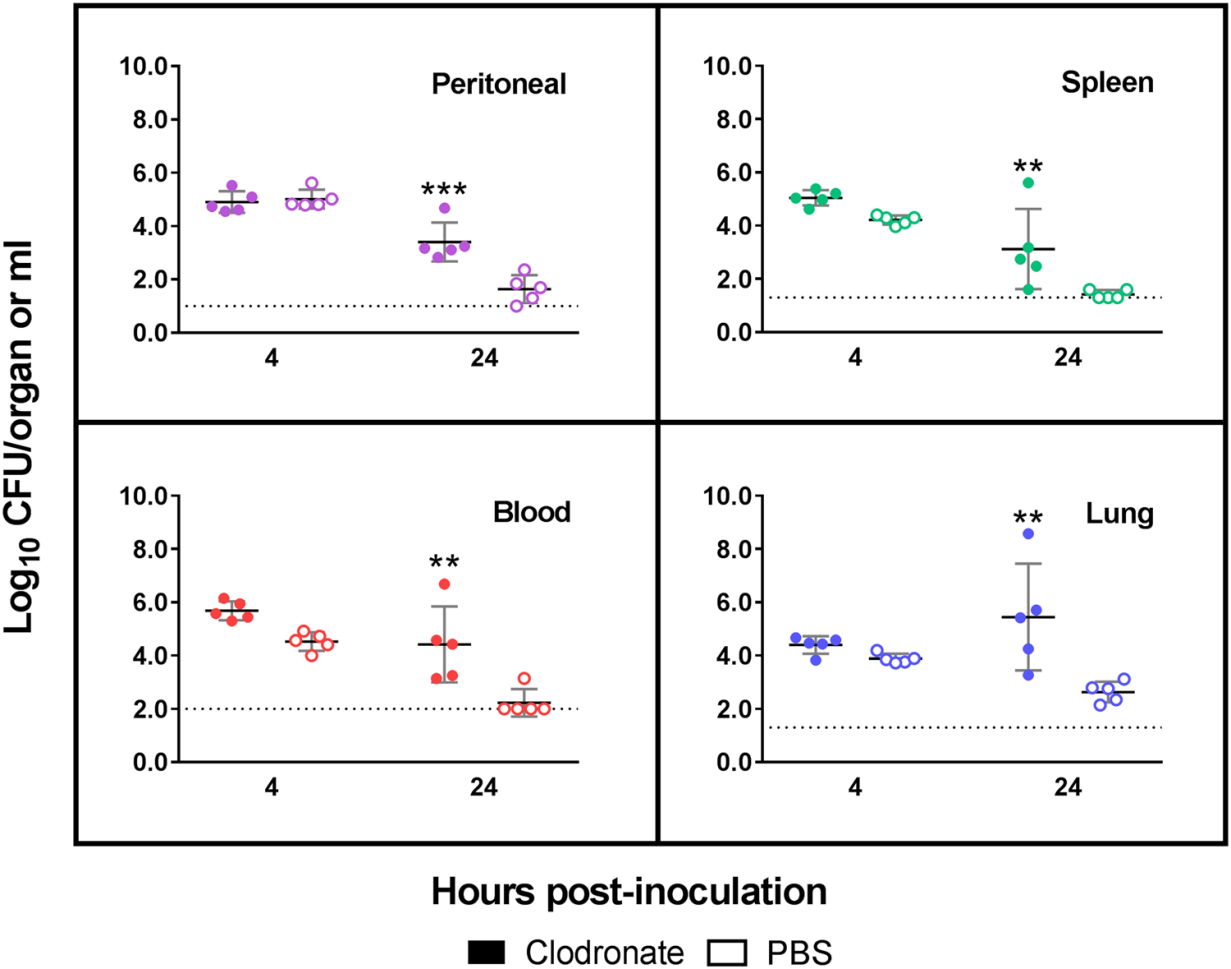
*In vivo* macrophage depletion enhances the host susceptibility to i.p. *A. baumannii* LAC-4 infection. Groups of C57BL/6 mice were i.p. treated with either clodronate-liposomes (clodronate) to deplete peritoneal macrophages or PBS-liposomes (PBS) as sham-depletion controls. Twenty-four hours later, all mice were i.p. inoculated with 4.8 × 10^4^ CFU freshly cultured *A. baumannii* LAC-4. Five mice from each group were euthanized at 4 and 24 h post-inoculation. The bacterial burdens in the peritoneal lavage fluid, blood, spleen, and lung were determined by quantitative bacteriology. Data from individual mouse are presented as scatter plot and the horizontal bar represents the mean (n = 5). **P < 0.01 and ***P < 0.001 (Two-way ANOVA with Sidak’s multiple comparisons test).

Results of the above experiments and from previous studies by other laboratories suggest that macrophages may play an important role in host defense against i.p. LAC-4 infection^15,19,24,25^. To obtain direct evidence of the contribution of macrophages in host defense against i.p. LAC-4 infection, groups of C57BL/6 mice were i.p. treated with clodronate-liposomes to deplete peritoneal macrophages (depleted mice) or PBS-liposomes as treatment control (control mice). Twenty-four hours later, the mice were i.p. inoculated with 4.8 × 10^4^ CFU LAC-4, and bacterial burdens in the peritoneal lavage fluid, blood, lung, and spleen were determined 4 and 24 h p.i. Compared to control mice, the bacterial burdens in the depleted mice at 4 h p.i. were generally comparable (peritoneal lavage fluid) or slightly higher (blood and other tissues) (Fig. 7). At 24 h p.i., significantly higher numbers of bacteria were recovered in all examined tissues of depleted mice (P < 0.001 for peritoneal lavage fluid and P < 0.01 for blood, spleen and lung, respectively) (Fig. 7). In addition, the bacterial burdens were generally reduced in the tissues and blood at 24 h p.i. as compared to 4 h p.i., with the exception of the lungs where the bacterial burdens were higher.

## Discussion

Septicemia is one of the major clinical manifestations of *A. baumannii* infection^3,4^. The mouse model of i.p. *A. baumannii* infection has been commonly used for infection pathogenesis studies and for anti-*A. baumannii* therapeutics and vaccines evaluation. However, there are surprisingly few studies on the characterization of this model^15,17,21,26^. In this study, we described a mouse model of i.p. *A. baumannii* infection using a hypervirulent strain and commonly used mouse strains, and characterized the innate cellular and proinflammatory cytokine responses to the infection. Our results shared several similarities with other published studies in which the innate immune responses to i.p. infection with a clinical *A. baumannii* strain have been examined in some details^15,17,26^. The infected mice either succumb to the infection very rapidly or completely recover from the infection within 1–2 days. These results suggest that the i.p. *A. baumannii* infection in mice appears to fit a “single-hit” infection model^27^. Similar to most *A. baumannii* clinical and type strains studied, the minimal dose of LAC-4 needed to cause a lethal i.p. infection (10^6^ CFU) is substantially lower than that of i.n. inoculation (~10^8^ CFU)^12^.

The reason for the observed hypervirulence of LAC-4 in mice remains unknown. The ability of rapid systemic dissemination by LAC-4 was originally postulated as a potential contributing factor for its high virulence in i.n. inoculation^12^. However, the systemic dissemination is irrelevant in i.v. infection^19^ and only play a limited role in i.p infection in this study, suggesting that other unidentified mechanisms may contribute to the hypervirulence of this strain.

To the best of our knowledge, LAC-4 is one of only a few reported clinical and type *A. baumannii* strains/isolates that are hypervirulent to commonly used immunocompetent mice (such as C57BL/6 and BALB/c mice) and which cause 100% mortality within 48 hours of inoculation^15,18–21,23,28,29^. Previous studies by others have generally used porcine mucin or immunocompromised mice (such as neutropenic or diabetic mice)^21,23,30–33^ to enhance bacterial virulence or host susceptibility, in order to ensure a successful infection. Alternatively, a large inoculation dose (up to 10^10^ CFU) is required to induce an infection in conventional mice^34^. The ability to use a low challenge inoculum and conventional mouse strains (such as BALB/c and C57BL/6) in the experimental model described here has several advantages over other models. These include the likely mimicry of human natural infection and a better model reproducibility. In addition, the presence of an intact immune system would allow for the study of the interaction between bacterial infection and host immune system, and for evaluation of immune-based anti-*A baumannii* therapeutics. Moreover, LAC-4 is a multidrug resistant strain and its genome is fully sequenced and annotated^18,22,35^. However, it should be noted that LAC-4 displays a genomic profile distinct from most of the other outbreak isolates reported to date, as well as from ATCC 17978, ATCC 19606 and AYE strains^22^; this may limit the utilization of this model to certain type of studies.

Results of the kinetics and tissue bacterial burden study suggest that LAC-4 possesses unique capabilities of rapid local replication and systemic dissemination to blood and other tissues. The presence of higher bacterial burdens than the initial inoculum at the site of inoculation (peritoneal cavity) and blood at 4 h p.i. is noteworthy because various *A. baumannii* strains/isolates reported to date generally do not replicate in immunocompetent mice^16,19–21,23,36,37^. The immediate, net increase in the local LAC-4 burden suggests that this strain is capable of evading the host innate immunity since the doubling time of this strain in *in vitro* culture is similar to other *A. baumannii* strains^19^. Our previous studies in the mouse model of i.n. infection also suggests that the host innate immunity appears to be less effective in controlling LAC-4 proliferation as compared to some ATCC strains or clinical isolates^18^.

Similar to i.n. *A. baumannii* infection in mice, i.p. LAC-4 infection induced a rapid recruitment of neutrophils and associated reduction in the percentages of peritoneal macrophages and lymphocytes (Fig. 3). We and others have shown that neutrophils and macrophages play important roles in host innate resistance to different routes of *A. baumannii* infection^23,31,38^. It has also been demonstrated that neutrophils, but not NK1.1^+^ cells, play a crucial role in host defense against i.p. *A. baumannii* infection^15^. However, the role of macrophages remains unknown. In this study, we found that the tissue and blood bacterial burdens in macrophage-depleted mice were significantly higher than macrophage-intact mice following i.p. LAC-4 challenge (Fig. 7), suggesting that macrophages also play an important role in host defense against i.p. *A. baumannii* infection^19,24^. In this regard, our preliminary FACS analysis of the subpopulation and activation of peritoneal cells (Fig. 4) indicates a substantial reduction in the surface expression of CD86 co-stimulation molecule and MHC-II by F4/80^+^ peritoneal macrophages at 24 h.p.i. This suggests that the LAC-4 is able to suppress the macrophage activation and its function, which may in turn contribute to the subsequent clinical outcome of the infected mice. On the other hand, the infection showed minimal effects on the composition of B or T lymphocytes with the exception that the proposition of γδT cells were significantly increased at 24 h.p.i. Since it is well recognized that the peritoneal macrophages are heterogeneous populations with distinct development and function and mount dynamic responses to infection^39^, further detailed profiling and characterization of this population of cells in response to *A. baumannii* will be needed.

As reported in other *A. baumannii* infection studies in mice and in human cell cultures, i.p. LAC-4 infection induced significant increases in the local and systemic levels of many different cytokines/chemokines (Fig. 5). It has been suggested that the cytokine/chemokine response observed in the infected mice reflects more the high bacterial burdens than it does the host innate immunity^23,30,32,33,40^. On the other hand, elevated levels (such as TNF-α) are generally not directly related to mortality either^40^.

We previously profiled the local (lung) and systemic (serum) cytokine/chemokine responses to i.n. LAC-4 infection in mice^18^. It appears that the cytokine/chemokine response to i.n. and i.p. infection with LAC-4 share a similar profile, in that the serum levels of TNF-α, RANTES, MIP-1β, MCP-1, KC, IL-12p40, IL-6, IL-1β, GM-CSF and anti-inflammatory cytokine IL-10 were significantly increased whereas the IL-17 level was significantly increased only in i.n., but not i.p., infected mice, perhaps reflecting the fact the double negative resident lung T cells are a major IL-17 producing source^41^. It is interesting to note that the cytokine/chemokine responses at the site of inoculation also shared many similarities, despite the distinctive anatomic location (peritoneal cavity for i.p. vs lung for i.n.); both routes of LAC-4 inoculation induced significantly increases in the level of IL-1α, IL-1β, IL-6, MIP-1β, MCP-1, and KC and, to a lesser magnitude, TNF-α and RANTES in the peritoneal or lung lavage fluid, respectively (Fig. 5). On the other hand, GM-CSF and IL-17 were increased only in the lung lavage fluid following i.n. LAC-4 infection^18^, likely reflecting the anatomical differences in the type of resident cytokine-producing cells and the regulatory pathway of these cytokines.

As anticipated, both quantitative and qualitative differences were observed in the cytokine/chemokine responses to different doses of i.p. LAC-4 inoculation (Fig. 5). There was a dose-related difference in the level of RANTES and IL-10 in the peritoneal lavage fluid, whereas increases in IFN-γ, IL-17, IL-4, IL-5 and IL-9 were only observed in the mice challenged with the high dose of LAC-4. On the other hand, the cytokine/chemokine changes in the serum were more dose-related. Those results suggest that the local and systemic cytokine/chemokine responses to *A. baumannii* in mice are largely related to the challenge strain and dose used and less influenced by the challenge route. Further studies and detailed analysis of the cytokine and chemokine responses will be needed to better understand their role in the infection pathogenesis and host defense.

As in i.p. LAC-4 infected mice, *in vitro* stimulation of peritoneal macrophages with ffLAC-4 induced high levels of TNF-α, RANTES, MIP-1β, MCP-1, KC, GM-CSF, IL-10, IL-6, IL-1α and IL-1β production in the culture supernatant (Fig. 6), suggesting that macrophages participate in the early inflammatory responses and host defense against i.p. LAC-4 infection and contribute to the initiation of a cascade of proinflammatory cytokine/chemokine production and innate immune cell recruitment.

In conclusion, our study has demonstrated that i.p. LAC-4 infection of immunocompetent C57BL/6 and BALB/c mice induces a lethal or sublethal infection, depending on the inoculation dose, with high bacterial burdens in peritoneal cavity, blood and tissues. The infection induces acute peritoneal recruitment of neutrophils and other innate immune cells, as well as the local and systemic production of proinflammatory cytokines and chemokines. This mouse infection model will provide an alternative and useful tool for future pathogenesis studies of *A. baumannii-*associated septicemia and identification and characterization of important virulence factors, and can serve as a surrogate model for rapid evaluation of novel therapeutics and vaccines for this emerging infectious agent.

## Methods

### Mice

Eight- to 12-wk-old specific-pathogen-free female C57BL/6 and BALB/c mice were purchased from Charles River Laboratories (St. Constant, Quebec). The mice were maintained and used in accordance with the recommendations of the Canadian Council on Animal Care Guide to the Care and Use of Experimental Animals. All experimental procedures were approved by the institutional animal care committee (Institute for Biological Sciences, National Research Council Canada).

### A. baumannii

*A. baumannii* ATCC 17961 (American Tissue Type Culture, Manassas, VA) and LAC-4 were used in the study. LAC-4 is a multidrug resistant clinical isolate originally from an endemic outbreak in a hospital at the Los Angeles County^42^ and has been demonstrated to be a hypervirulent isolate in mice by i.n. or i.v. inoculation^18,19^.

### Intraperitoneal *A. baumannii* inoculation and sample collections

For i.p. inoculation in mice, freshly cultured inocula were prepared for each experiment from frozen stocks of *A. baumannii* as previously described^23^. Mice were inoculated with indicated numbers of *A. baumannii* in 100 µl saline using a 1.0 ml tuberculin syringe with a 30 G needle. Actual inocula in each experiment were determined by plating 10-fold serial dilutions on brain-heart infusion (BHI) agar plates. The clinical appearance of the mice was monitored and scored as described previously^23^. Groups of three to five infected mice were sacrificed at pre-determined time points post inoculation. The peritoneal cavity was lavaged with 10 ml of phosphate buffered saline (PBS) supplemented with 3 mM Ethylenediaminetetraacetic acid (EDTA) and 1% fetal bovine sera (FBS). Blood samples were collected for bacterial culture or serum separation. The lungs, spleens, and kidneys were aseptically removed and used for quantitative bacteriology.

### Quantitative bacteriology

The lungs, spleen and kidneys were homogenized in sterile saline using aerosol-proof homogenizers. Aliquots (100 µl) of 10-fold serial dilutions of the tissue homogenates, whole blood, and peritoneal lavage fluid were cultured on BHI agar plates to quantify the number of viable *A. baumannii* in the respective samples^23^.

### Quantification and FACS analysis of peritoneal lavage cells

The total number of peritoneal lavage cells was determined with a hemacytometer and differential cell counts were determined on cytospin slides (Cytospin 3, Shandon, Pittsburgh, PA) stained with Hema-3 (Fisher Scientific, Kalamazoo, MI)^18^. The lavage fluid was centrifuged at 2,450 × g for 7 min, and the supernatant collected, filter-sterilized and stored at −20 °C.

The percentage and activation of some peritoneal cell populations were also determined by FACS analysis as described previously^24^ and in Supplementary Methods. Briefly, the cells were washed in PBS containing 1% BSA, and incubated with unlabeled anti-CD16/CD32 (clone 2.4G2) monoclonal antibodies (BD Biosciences, San Jose, CA) for 15 min to block non-specific Fc receptor binding. Aliquots containing ~10^6^ cells were stained with antibody cocktails containing appropriate fluorochrome-conjugated mAb for 30 min at 4 °C. The anti-F4/80 mAb was used as a marker for macrophages and the activation of F4/80^+^ macrophages was further analyzed using anti-CD80 (clone 16–10A1), anti-CD86 (clone GL1), anti-CD40 (clone 3/23), and anti-MHCII (AF6-120.1) antibodies. After staining, the cells were washed twice with the above PBS/BSA solution, fixed with 200 μl of 1% paraformaldehyde (Polysciences Inc., Warrington, PA), and stored in the dark at 4 °C until ready for analysis. The data were acquired using a FACS Canto flow cytometer (BD Biosciences) and analyzed using FlowJo software (Tree Star, Inc., Ashland, OR).

### Production of cytokines and chemokines by *A. baumannii*-stimulated peritoneal macrophages

In some experiments, the peritoneal lavage cells from uninfected mice were harvested. One ml aliquots of the cells (5 × 10^5^ cells/ml) were seeded into 24-well tissue culture plates (Becton Dickinson, Mississauga ON), and stimulated with 5 × 10^7^ formalin-fixed LAC-4 cells (ffLAC-4) or media only as controls^43^. Culture supernatants were harvested at 24 or 48 h after the addition of ffLAC-4, centrifuged, and stored at −20 °C until assay.

### Cytokine and chemokine assays

Levels of cytokines and chemokines in the sera, peritoneal lavage fluid, and cell culture supernatant were measured using 21-plex Milliplex MAP mouse cytokine/chemokine kits (Millipore, Ltd. Billerica, MA) on a Luminex 100IS system (Luminex, Austin, TX), as specified by the manufacturer. Samples were assayed in duplicate, and cytokine/chemokine concentrations were calculated against the standards using Beadview software (ver 1.03, Upstate)^23^.

### *In vivo* macrophage depletion

Peritoneal macrophages were depleted by i.p. administration of liposomes encapsulated dichloromethylene diphosphonate (clodronate, or CL_2_MDP). Liposomes containing clodronate (clodronate-liposomes) and liposomes encapsulating PBS (PBS-liposomes) were purchased from Liposoma BV (Amsterdam, Netherland). Groups of five C57BL/6 mice were i.p. injected with 100 µl of clodronate-liposomes or PBS-liposomes at 24 and 4 h prior to infection. Mice were then inoculated i.p. with 5 × 10^4^ CFU LAC-4 as described above. Infected mice were sacrificed at 4 and 24 h p.i., and the blood, peritoneal lavage fluid, lungs, and spleens were aseptically collected, and used for quantitative bacteriology^23^.

### Statistical analysis

Data are presented as means ±SD for each group, unless otherwise specified. Differences in quantitative measurements were assessed by Student’s *t* test, one-way or two-way analysis of variance (ANOVA) followed by Bonferroni’s *post-hoc* multiple comparison tests, when appropriate. Differences were considered significant when P < 0.05.

## Supplementary information

Supplementary Info

## Data Availability

The datasets generated during and/or analysed during the current study are available from the corresponding author on reasonable request.
