# Supplementary material for: Acute intraperitoneal infection with a hypervirulent Acinetobacter baumannii isolate in mice
Source: Sci Rep. 2019 Apr 25;9:6538. doi: 10.1038/s41598-019-43000-4 (PMC6484084; doi:10.1038/s41598-019-43000-4)
Supplement: Supplementary file 1 — Supplementary Info [file 41598_2019_43000_MOESM1_ESM.pdf]

Supplementary Information

Acute intraperitoneal infection with a hypervirulent *Acinetobacter baumannii* isolate in mice

Greg Harris, Rhonda KuoLee, H. Howard Xu, and Wangxue Chen

Methods:

FACS analysis gating strategy

Macrophages/granulocytes: Peritoneal cells were gated by forward and side scatter, then by F4/80-PerCP-Cy5.5, CD11b-APC, CD11C-APC-Cy7 and Gr-1-FITC.

Macrophage activation: Peritoneal cells were gated by forward and side scatter, then by F4/80-APC, then by CD40-FITC, CD80-PerCP-Cy5.5, CD86-PE-Cy7 and MHC-II-PE

Lymphocytes, T cell subsets: Peritoneal cells were gated by forward and side scatter, then by CD19-APC and CD3-PE-Cy7. $CD3^+$ cells were then gated by TCRβ-FITC and TCRγδ-PE

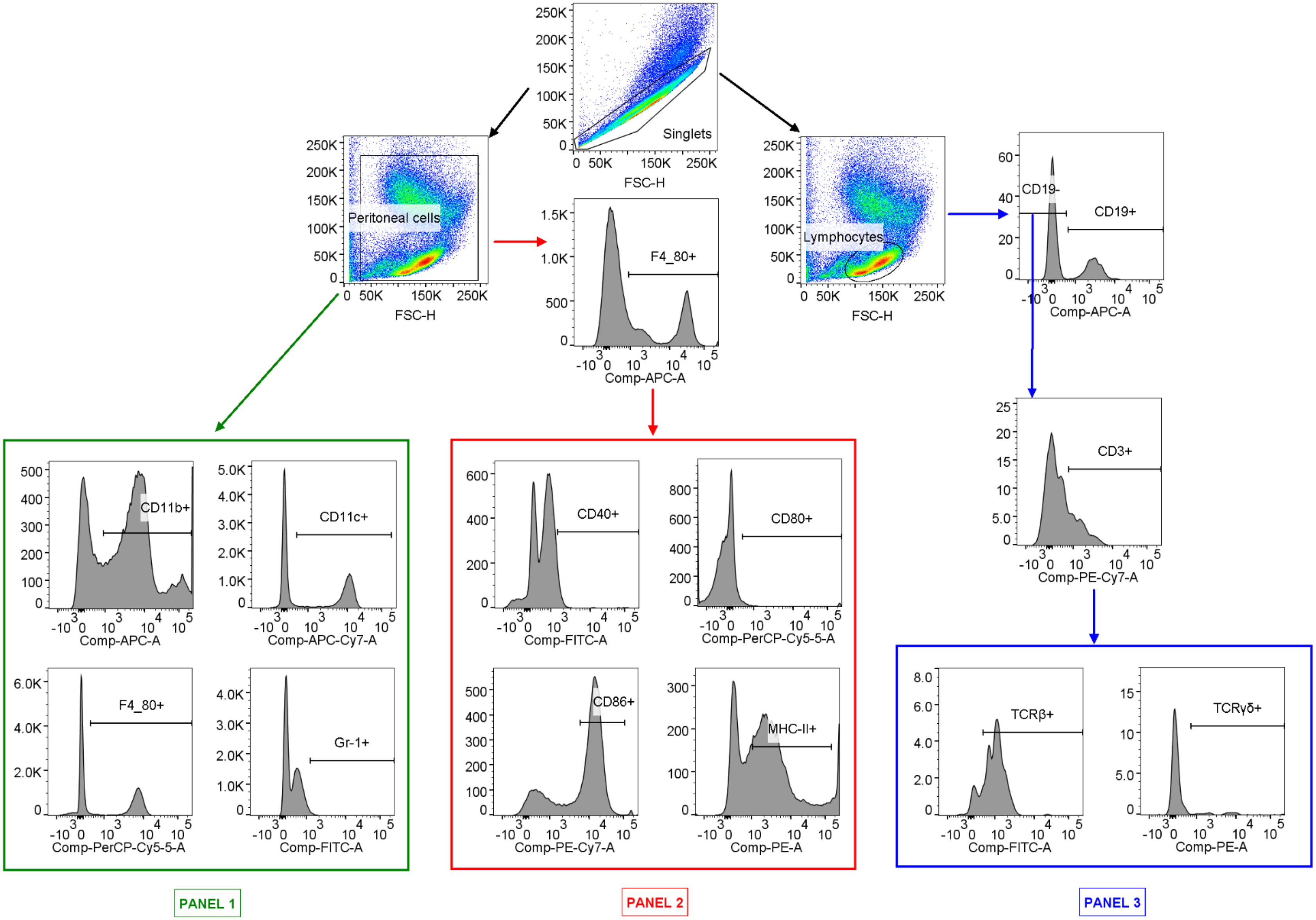
Singlets
FSC-H
Peritoneal cells
FSC-H
F4_80+
Comp-APC-A
Lymphocytes
FSC-H
CD19-
CD19+
Comp-APC-A
CD3+
Comp-PE-Cy7-A
CD11b+
Comp-APC-A
CD11c+
Comp-APC-Cy7-A
F4_80+
Comp-PerCP-Cy5-5-A
Gr-1+
Comp-FITC-A
CD40+
Comp-FITC-A
CD80+
Comp-PerCP-Cy5-5-A
CD86+
Comp-PE-Cy7-A
MHC-II+
Comp-PE-A
TCRβ+
Comp-FITC-A
TCRγδ+
Comp-PE-A
PANEL 1
PANEL 2
PANEL 3

Figure

Fig. S1 Photomicrographs of peritoneal cells from mice killed at the indicated time following the i.p. inoculation with LAC-4. (A) Peritoneal cells from a mouse killed at 0 h, showing the presence of predominantly resting macrophages, lymphocytes and mast cells without the presence of neutrophils; (B) Peritoneal cells from a lethally infected mouse killed at 4 h p.i., showing the presence of macrophages and lymphocytes admixed with moderate numbers of neutrophils; (C) Peritoneal cells from a sublethally infected mouse killed at 4 h p.i., showing predominantly neutrophils with horseshoe-shaped nuclei admixed with small numbers of macrophages or lymphocytes; (D) Peritoneal cells from a sublethally infected mouse killed at 24 h p.i., consisting of almost entirely neutrophils with occasional degenerated macrophages; (E) Peritoneal cells from a sublethally infected mouse killed at 48 h p.i., showing that neutrophils remain the predominant cell subpopulation although their proportion is reduced as compared to 24 h p.i. ; (F) Peritoneal cells from a sublethally infected mouse killed at 72 h p.i., showing the significant reduction in neutrophils and increases in the proportion of macrophages or lymphocytes; (G) Peritoneal cells from a mouse killed at 168 h p.i., showing predominantly macrophages and lymphocytes admixed with small numbers of neutrophils. Hema-3 staining, bar = 100 μm. Black arrows: macrophages; white arrows: lymphocytes; arrowheads: neutrophils.

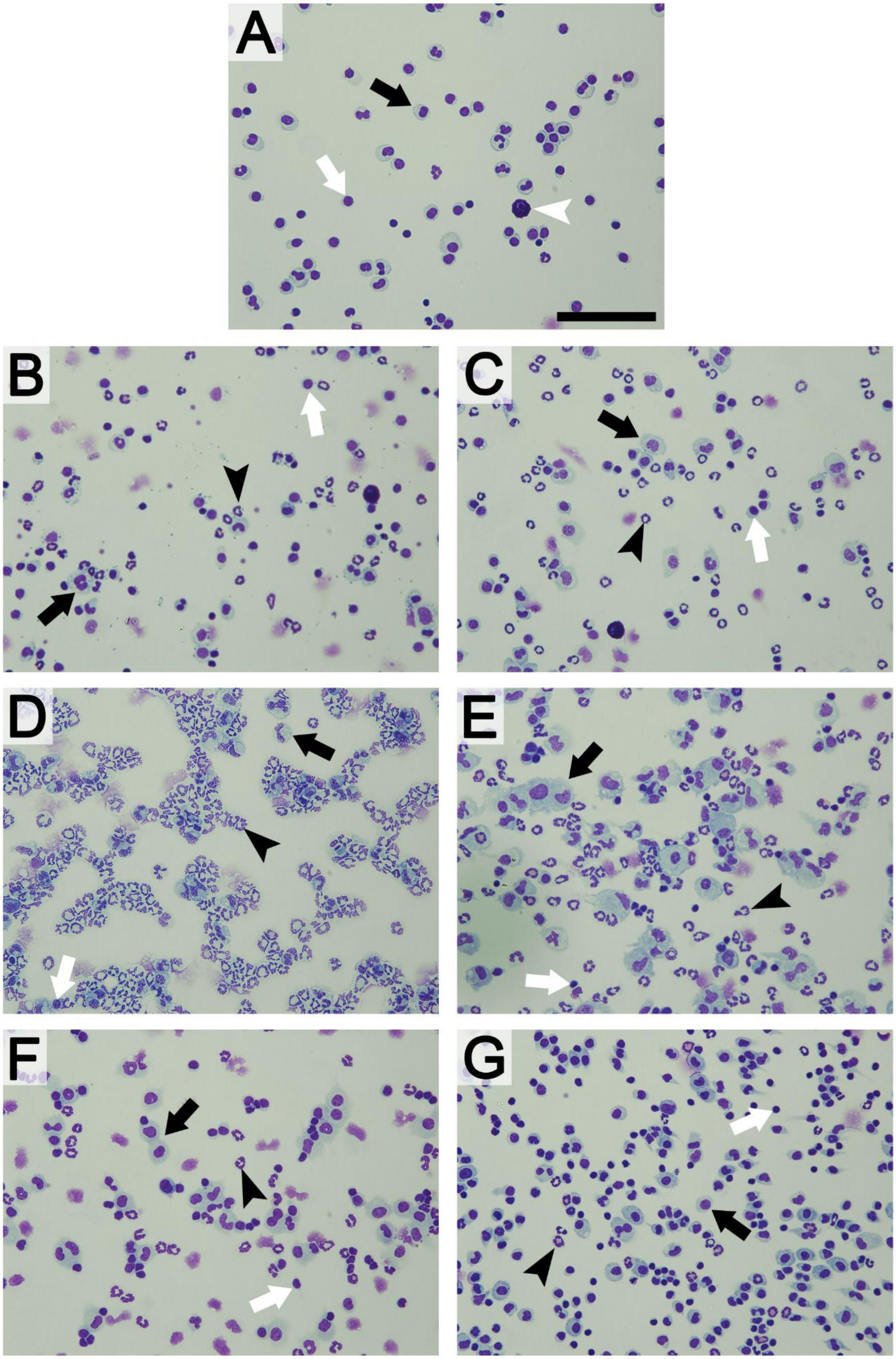
A
B
C
D
E
F
G
